# Use of a digital rescue inhaler and at-home spirometer among inner-city children with asthma: a real-world experience

**DOI:** 10.3389/falgy.2025.1641312

**Published:** 2025-09-22

**Authors:** Neema Izadi, Tanisha D. Hill, Amanda Boe, Daisy Yu, Jonathan S. Tam

**Affiliations:** 1 Division of Clinical Immunology & Allergy, Children’s Hospital Los Angeles, Los Angeles, CA, United States; 2 Teva Branded Pharmaceutical Products R&D, Inc., Parsippany, NJ, United States

**Keywords:** inner-city, underserved, asthma, pediatrics, adherence, digital inhaler, spirometry, rescue inhaler

## Abstract

**Background:**

Across all age groups, asthma disproportionally affects inner-city underserved populations. Studies on the use of at-home spirometry and digital inhalers have limited real-world evaluation in pediatric asthma.

**Objectives:**

In this prospective exploratory study, we assessed how an integrated digital rescue inhaler and at-home spirometer would affect proper inhaler use, medication adherence, and asthma outcomes using a minimalistic real-world approach.

**Methods:**

In total, 21 pediatric patients with asthma (8–17 years of age) were asked to replace rescue medications with the ProAir Digihaler and perform at-home gamified spirometry daily. Lung function and questionnaires were obtained at baseline and at 3–4 months.

**Results:**

The participants were mostly male (81%), Latino/Hispanic (71%), and obese (88th ±16 percentile). Proper rescue inhaler step identification by survey did not change, but inhalation technique based on digital inhaler flow measurements improved for all participants. At-home spirometry was sporadic and reported controller adherence did not change. Younger children (age 8–11) were more severe at baseline [Composite Asthma Severity Index (CASI) of 4.8] compared to older children (CASI of 2.9). For younger children, overall asthma control test scores increased by 3.1, CASI decreased by 0.70, and the Pediatric Quality of Life Inventory scores increased by 14 and 11 for participants and parents, respectively.

**Conclusions:**

Proper rescue inhaler step identification by survey did not change, but actual inhalation technique based on digital inhaler flow measurements improved. At-home spirometry was sporadic and reported medication adherence did not change. Younger children used the spirometer more frequently and demonstrated improvements in asthma control, severity, and quality of life. These improvements were not observed in older children.

## Introduction

Poorly controlled asthma leads to major disability, financial burden, and reduced quality of life (1, 2). Across all age groups, asthma disproportionally affects underserved populations of lower income (3), with a high burden among inner-city children (4). Non-adherence to asthma medications leads to markedly increased morbidity, mortality, and costs (2, 5–8). Patients and their families commonly over-report adherence to be looked upon favorably by their physician, otherwise known as social desirability bias (9). Recent data also suggests that a majority of patients are deficient in technique for the appropriate use of both rescue and controller medications (10). Digital inhalers can improve asthma outcomes (11–13), but are rarely used in clinical practice due to availability, added costs, and poor clinician detection of non-adherent patients (14).

One of the first digital inhaler devices came in the form of an albuterol dry-powder inhaler (DPI) with an embedded sensor that detects and records breath-activated inhalations of the device along with inspiratory flow rate in liters per minute (ProAir® Digihaler™ by Teva Pharmaceutical Industries Ltd., Waterford, Ireland). Notably, the Digihaler application informs participants of inhalation “quality” using a simple labeling system of good (green), fair (yellow), and poor (red) based on flow rate. Furthermore, the Digihaler application also offers features to track inhaler use and set reminders. Studies have shown that the use of digital inhalers may not only improve adherence, but may also have a positive impact on appropriate inhaler use technique in clinical practice (10). In addition, a recent Markov model-based cost-utility analysis demonstrated that digital inhaler-based interventions can lead to cost-saving by optimizing inhaler adherence, technique, and reducing the need for biologics (15).

In this exploratory pilot study, we evaluated pediatric patients with asthma from a large tertiary hospital that serves a mostly underserved inner-city population. We assessed whether replacing a conventional rescue inhaler with the ProAir Digihaler could improve technique by inhalation quality and step identification, and whether patients would utilize a gamified at-home spirometer and how this may affect controller adherence. We also evaluated additional parameters, including asthma control, quality of life, risk parameters, and lung function, to explore any changes in asthma outcomes. Compared to previous studies, we used a minimalistic real-world approach without additional reminders, interventions, or reward systems that may not be feasible in a busy practice or when applied to larger populations. Our goal was to assess rescue use and home spirometry rather than controller adherence, which has already been extensively studied.

## Methods

### Study population

In total, 21 children aged 8–17 were enrolled from the Allergy Specialty Clinic at Children's Hospital Los Angeles (CHLA) for a prospective 3–4-month single-arm non-randomized study. The participants were required to be at least 8 years of age to optimize the use of the devices, including the Aluna at-home gamified spirometer. The patients were required to have asthma diagnosed by an allergist and albuterol already prescribed. The exclusion criteria included any chronic lung disease other than asthma or significant comorbidities that affect lung function.

### Study design

Participants were seen for an initial visit and a post-visit approximately 3–4 months later. Study data were collected and managed using the Research Electronic Data Capture (REDCap) (16, 17) electronic data capture tools hosted at CHLA. Participants completed REDCap questionnaires at the initial visit on demographic and medical history information, and at both visits for asthma history, albuterol use and technique, Test of Adherence to Inhalers (TAI)-10 Item (18), Composite Asthma Severity Index (CASI) (19, 20), Asthma Control Test (ACT) (21), and the Pediatric Quality of Life Inventory (PedsQL) Asthma Module 3.0 (22). All the questionnaires were completed by the child and/or parent based on the specific age restrictions and embedded requirements of the questionnaire. The proper use of albuterol was determined based on detailed questions on inhaler technique and peak inspiratory flow rates measured while using the ProAir Digihaler. Nebulizer technique was also assessed, but not enough participants chose this modality for analysis. Peak expiratory flow (PEF) and forced expiratory volume in the first second (FEV_1_) were recorded in triplicate at both visits via the Microlife PF 100 Peak Flow Meter. At the initial visit, participants were given three ProAir Digihalers and asked to use these in place of their rescue medications.

### Digihaler and Aluna spirometer implementation

The ProAir Digihaler application was installed on one designated smartphone based on family preference. The details of the digital system used with the ProAir Digihaler have been previously described (13). A brief demonstration of the ProAir Digihaler was provided at the initial visit. The setup of the Digihaler application was done with the patient at the initial visit, but no features were demonstrated, and use was optional. The physician dashboard was not monitored, and no additional interventions were implemented. The participants were also given an Aluna at-home gamified spirometer and asked to use it at least once daily. The technique for the Aluna spirometer was indirectly practiced when the participants used the Microlife PF 100 Peak Flow Meter. The Aluna spirometer application was installed on the same smartphone and no features were demonstrated.

### Statistics

This was a non-interventional, observational study. All the endpoints and variables were summarized using descriptive statistics. The descriptive statistics for the continuous variables included the number of patients (*n*), mean, standard deviation (SD), median, and range. The descriptive statistics for the categorical variables included observed responses in each category and percentages. Given its single-arm, exploratory nature and the constraints of a small sample size, this pilot study did not include formal hypothesis testing.

## Results

### Characteristics of the study population

The participants included 21 children aged 8–17 with asthma from the Allergy Specialty Clinic at CHLA. There were, on average, 109 (±21) days between the baseline and post-visit (Table 1). One participant did not complete the post-visit. The participants were mostly male (81%) and identified as Latino/Hispanic (71%). Most patients were obese and had other atopic conditions, but did not declare any depression or anxiety. The participants did not report any first- or second-degree smoke exposure of any kind. A little over half of the parents had asthma, and the highest parental education level varied, with most having some college education or a bachelor's degree.

**Table 1:** Characteristics of the participants at baseline, *n* = 21 [data presented as *n* (%) or mean (SD)].

Characteristic	Baseline
Age (years)	12.6 (2.7)
Gender (male)	17 (81%)
Body mass index (kg/m^2^) percentile	88th (16)
Age at symptom onset (years)	4.0 (3.4)
Age at MD diagnosis (years)	4.5 (3.3)
Exposed to smoke in the household	0 (%)
Parental asthma	12 (57%)
Race	
African American	2 (10%)
Asian	1 (5%)
Caucasian	1 (5%)
Latino/Hispanic	15 (71%)
Multiple	2 (10%)
Comorbidities	
Eczema	11 (52%)
Allergic rhinitis or hay fever	4 (19%)
Food allergy	6 (29%)
Reflux	0 (%)
Depression	0 (%)
Anxiety	0 (%)
Highest parental education	
Less than high school completion	2 (10%)
High school graduate	3 (14%)
Some college education	7 (33%)
Associate or bachelor's degree	7 (33%)
Master's degree	2 (10%)

### Digihaler use and quality of inhalations

**Figure 1:**
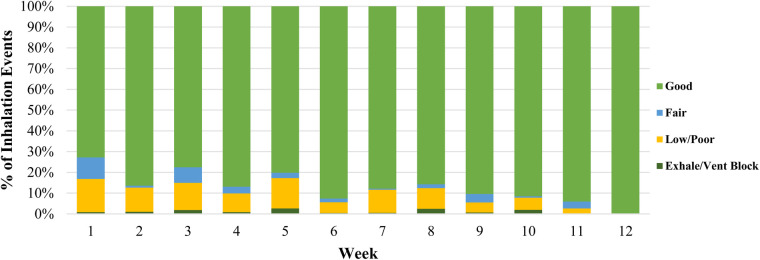
Quality of Digihaler rescue inhalations over time.

Rescue uses with the digital inhaler varied widely among participants (Supplementary Table S1). Younger children demonstrated fewer rescue uses per week (Table 2). The provided ProAir Digihalers were the only rescue inhalers used by almost all participants during the study period based on the post-visit questionnaire (Supplementary Table S1). On average, the quality of inhalations gradually improved over time from 83% fair/good inhalations to 100% fair/good inhalations (Figure 1).

**Table 2:** Outcome measures of the participants at baseline and the 3−4-month post-visit [data presented as mean (SD) unless otherwise specified].

Measure	Younger children		Older children		All participants	
	Age 8–11		Age 12–17		Age 8–17	
	Baseline	Post	Baseline	Post	Baseline	Post
Participants (*n*)	8	7	13	13	21	20
Asthma control						
ACT score	20.0 (1.9)	23.1 (±2.4)	21.2 (3.1)	20.7 (4.0)	20.8 (2.8)	21.6 (3.7)
	Median: 20.0	Median: 24.0	Median: 22.0	Median: 22.0	Median: 21.0	Median: 22.0
	Range: 17–22	Range: 19–26	Range: 15–25	Range: 12–25	Range: 15–25	Range: 12–26
CASI score	4.8 (3.5)	3.6 (2.6)	2.9 (2.4)	3.2 (3.1)	3.6 (2.9)	3.4 (2.8)
	Median: 5.5	Median: 4.0	Median: 3.0	Median: 3.0	Median: 3.0	Median: 3.0
	Range: 1–8	Range: 1–7	Range: 0–9	Range: 0–11	Range: 0–9	Range: 0–11
Lung function^a^						
FEV_1_ (L)	1.8 (0.4)	1.8 (0.3)	2.7 (0.8)	2.9 (0.7)	2.4 (0.8)	2.6 (0.8)
	Median: 1.8	Median: 1.7	Median: 2.7	Median: 2.9	Median: 2.4	Median: 2.5
	Range: 1.3–2.5	Range: 1.5–2.4	Range: 1.3–3.7	Range: 1.9–4.1	Range: 1.3–3.7	Range: 1.5–4.1
FEV_1_% predicted	97.1 (13.9)	99.4 (10.5)	94.9 (22.6)	100.3 (15.9)	95.7 (19.3)	99.9 (13.9)
	Median: 94.0	Median: 94.2	Median: 97.7	Median: 96.4	Median: 95.9	Median: 96.0
	Range: 76–118	Range: 90–116	Range: 37–127	Range: 77–132	Range: 37–127	Range: 77–132
PEF (L/min)	271 (55)	257 (53)	367 (104)	415 (98)	331 (99)	360 (114)
	Median: 270	Median: 259	Median: 367	Median: 413	Median: 323	Median: 338
	Range: 184–369	Range: 179–353	Range: 199–551	Range: 270–536	Range: 184–551	Range: 179–536
Quality of life						
PedsQL Child	67.3 (13.2)	82.1 (8.6)	86.5 (12.3)	86.7 (10.4)	81.7 (16.8)	87.8 (8.5)
	Median: 70.5	Median: 85.7	Median: 91.1	Median: 89.3	Median: 87.7	Median: 88.8
	Range: 47–84	Range: 70–93	Range: 55–100	Range: 65–100	Range: 45–100	Range: 67–100
PedsQL Parent	66.3 (11.8)	77.3 (9.4)	84.6 (14.0)	81.8 (11.5)	80.9 (15.6)	83.5 (9.8)
	Median: 67.4	Median: 75.9	Median: 86.6	Median: 79.5	Median: 80.1	Median: 84.4
	Range: 45–79	Range: 66–93	Range: 54–100	Range: 64–100	Range: 42–100	Range: 67–100
Adherence						
TAI score	45.3 (4.2)	43.1 (3.8)	44.6 (4.7)	44.8 (5.1)	44.9 (4.4)	44.2 (4.7)
	Median: 46.0	Median: 42.0	Median: 45.0	Median: 46.0	Median: 46.0	Median: 44.5
	Range: 36–50	Range: 38–50	Range: 36–50	Range: 33–50	Range: 36–50	Range: 33–50
Digihaler inhalations^b^						
Participants (*n*)^c^	8		11		19	
Inhalations per week	5.6 (9.0)		8.1 (10.6)		7.1 (9.8)	
	Median: 1.1		Median: 2.1		Median: 2.0	
	Range: 0.1–23.9		Range: 0.3–29.1		Range: 0.1–29.1	
Rescue-free days	8.1 (10.6)		8.1 (10.6)		8.1 (10.6)	
	Median: 2.1		Median: 2.1		Median: 2.1	
	Range: 0.3–29.1		Range: 0.3–29.1		Range: 0.3–29.1	
At-home spirometry						
Participants (*n*)	8		13		21	
Total spirometry uses	53 (56)		30 (54)		39 (54)	
	Median: 28		Median: 10		Median: 11	
	Range: 0–126		Range: 0–192		Range: 0–192	
Spirometry uses per day	30 (54)		30 (54)		30 (54)	
	Median: 10		Median: 10		Median: 10	
	Range: 0–192		Range: 0–192		Range: 0–192	

ACT, Asthma Control Test; CASI, Composite Asthma Severity Index; FEV_1_, forced expiratory volume in one second; PedsQL, Pediatric Quality of Life Inventory version 3.0; PEF, peak expiratory flow; TAI, Test of Adherence of Inhalers.

a Average of best three trials at the visit.

b Fair/good inhalations.

c No record (participants 15 and 16) or no post-visit (participant 12) for Digihaler inhalations.

### Proper rescue inhaler step identification by survey

Using a questionnaire, the participants were asked to identify the proper steps for the rescue inhaler technique, with different questions depending on inhaler type. Proper step identification was moderate at best, with no significant change at the post-visit and many responses left blank (Supplementary Table S2). Only two participants indicated that they used dry powder inhalers at baseline, with an overall 64% of the correct steps identified. Surprisingly, despite all being on the digital inhaler for the study, only nine participants were able to identify they were on a dry powder inhaler at the post-visit, with an overall 54% of the correct steps identified. Moreover, 12 participants used their rescue inhaler for pretreatment before exercise, and approximately half of them indicated the correct timing for such use at both the baseline and post-visit.

### At-home spirometer use and reported controller adherence

The Aluna at-home gamified spirometer was used by 19 of the 21 participants (Supplementary Table S3). Over half of the participants used it fewer than 20 times over the course of the study. The mean total number of spirometer uses was 39 (±54), and only three participants used the gamified spirometer at least once per day as requested. The overall average spirometer use per study day was 0.38 (±0.51). Based on the 10-item TAI, the participants remained borderline non-adherent to their controller medication, with TAI scores <45 at baseline (44.9±4.4) and at the post-visit (44.2±4.7) (Table 2).

### Subjective asthma control pre- and post-visit

Asthma control, as measured by the ACT, demonstrated borderline subjective control at baseline with an average score of 20.8 (±2.8) and no significant change in ACT score at the post-visit (21.6 ± 3.7; Table 2). However, the ACT score in the younger children (age 8–11) improved from 20.0 ± 1.9 to 23.1 ± 2.4, an overall change of 3.1, which was greater than the minimally important differences (MIDs) of 3 in adults (23) and 2 in children (24). Severity calculated by the CASI showed an average total CASI score of 3.6 (±2.9), indicating mild to moderate asthma at baseline, with no significant change at the post-visit (3.4±2.8). The younger children (age 8–11) had a more severe average baseline CASI score of 4.8 (±3.5), decreasing by 0.70 to 3.6 (±2.6) at the post-visit, which was greater than the minimally important difference of 0.49 for the CASI (20). In contrast, the older children (age 12–17) had milder asthma with an average CASI score of 2.9 (±2.4) at baseline and no significant change at the post-visit (3.2 ± 3.1). Notably, overall asthma controller medications decreased for nine participants over the course of the study, increased for four participants, and stayed the same for seven participants (Supplementary Table S4). During the study period, there were no hospitalizations and limited asthma risk to make significant inferences: five participants reported two ER and/or urgent care visits, four participants missed 1 day of school, three participants missed 2 days of school, and one participant missed 12 days of school (Supplementary Table S4).

### Lung function pre- and post-visit

Lung function improved slightly from baseline to post-visit (Table 2). PEF increased from 331 L/min (±99) to 360 L/min (±114). FEV_1_ increased from 2.4 L (±0.8) to 2.6 L (±0.8) and percent predicted FEV_1_ (pp FEV_1_) increased from 95.7% (±19.3) to 99.9% (±13.9). The older children were the primary driver of the lung function increases. Minimally important differences for lung function in pediatric asthma trials have not been well established. In adults and adolescents, meaningful change in ppFEV_1_ for asthma trials has been cited to be from 5% to 20% (24, 25).

### Quality of life pre- and post-visit

Quality of life based on the PedsQL improved in all domains from an overall score of 81.7 (±16.8) at baseline to 87.8 (±8.5) at the post-visit for the participants and 80.9 (±15.6) to 83.5 (±9.8) for their parents (Table 2). When stratified by age, the PedsQL score improved substantially for younger children from 67.3 (±13.2) to 82.1 (±8.6) for the participants and from 66.3 (±11.8) to 77.3 (±9.4) for their parents. These increases are well above the average 6.6 overall increase observed in larger samples of children with clinical asthma improvement over a similar timeframe using the same version (26). For older children, there was no change in the PedsQL score for the participants and a decrease for their parents.

## Discussion

Over the 3–4-month study period, almost all participants used the study-provided ProAir Digihaler as their only rescue inhaler with a wide range of inhalations per week. Although proper step identification of rescue inhaler technique via survey did not improve, the actual inhalation technique based on Digihaler flow measurements improved for all participants. This suggests that the improvements provided by the Digihaler were due to habit formation from real-time feedback—an approach that may be more effective than traditional methods that rely on memorization without built-in reinforcement.

The Aluna at-home gamified spirometer was not used consistently by most participants. Although well-received by our pediatric cohort, only three participants used it at least once daily as requested, and two participants did not use it at all. Reported adherence to medications did not change based on the TAI. Other limited studies on pediatric at-home spirometry have demonstrated mixed results for adherence and concordance with disease activity (27, 28). However, the younger children in our study used the spirometer more frequently and had improvements in subjective asthma control (ACT), severity (CASI), and quality of life (PedsQL). This discrepancy may be explained by different burdens of disease and oversight. Younger children had more severe asthma, with a lower quality of life at baseline for both the children and parents. This suggests that the additional monitoring provided by the digital rescue inhaler and at-home spirometer may benefit more motivated patients with more debilitating disease. While not directly studied, we imagine there was also more parental oversight for younger children to perform at-home spirometry and take advantage of both the Aluna and ProAir Digihaler cellphone applications. The rocket ship game in the Aluna spirometry application also appeals to a younger audience. Surprisingly, even though they had more severe asthma, the younger children had fewer rescue inhalations per week. We postulate that increased at-home spirometry for younger participants improved their understanding of asthma symptoms and reduced inappropriate rescue inhaler use. Future studies could add age-matched games to increase adherence in older children to investigate this possibility further.

The participants in this study were predominantly obese Hispanic boys with borderline subjective control and mild to moderate asthma. Obese children have a lower quality of life and worse asthma control (29). In addition, inner-city asthma in children is complex, with unique factors that increase asthma burden, such as social inequalities, housing quality, and poor access to care. Even after adjusting for neighborhood socioeconomic disparities, Black and Hispanic children have a higher incidence of asthma (30). Taken together, we studied a more severe and underrepresented demographic, and our findings may not apply to other populations.

Real-world evaluation of digital inhalers in pediatric asthma has been limited in underserved populations. In a study of 12 African American patients with asthma aged 11–16 years, a digital controller medication inhaler led to improved ACT and decreased rescue inhaler use over the 8-week study period (11). Notably, the digital inhaler system was built by investigators to support low literacy populations and included motivational interviewing and a monetary reward system. Another study of 14 non-Hispanic Black children with asthma and frequent exacerbations also found improvement in ACT over a 3-month study period with both digital rescue and digital controller inhalers (31). However, even with outreach by community healthcare workers for predefined alerts, they still found some feasibility concerns in recruitment, data transmission failure, and lost devices.

Overall, the participants demonstrated an increase in lung function, with the older children driving these findings. Given that the participants had no change in reported adherence and more had an overall decrease in asthma controller medications, these improvements were less likely due to increased controller use. However, these findings require validation with a larger sample size and longer study duration.

There were limitations to our pilot study. Recruitment was difficult for the target population, especially during the COVID-19 pandemic, leaving us with a smaller group to evaluate. Study duration was 3–4 months, but given the continued enrollment throughout the year, all seasons and school periods were represented. We found several barriers similar to those found by a previous pediatric digital asthma inhaler study by Kenyon et al. (31). Even with the setup of devices and applications during our initial study visit, the participants irregularly synced the ProAir Digihaler at home. Data transmission failures were rare, but some still occurred, requiring additional troubleshooting. Despite these barriers, it was reassuring to still observe some benefits from real-world digital inhaler rescue use and at-home spirometry in a mostly underserved population with high disease burden.

## Conclusion

Digital inhaler studies have historically included additional interventions, such as reminder phone calls or reward systems, that may not be feasible in a busy practice with limited resources. This is the first study of its kind to evaluate the use of FDA-approved integrated digital rescue inhalers in a pediatric underserved inner-city population with minimal researcher intervention, allowing for observation of real-world use. Proper rescue inhaler step identification by survey did not change, but actual inhalation technique based on digital inhaler flow measurements improved. At-home spirometry was sporadic and reported medication adherence did not change. However, the younger children, aged 8–11, demonstrated short-term benefits in asthma control, severity, and quality of life. These improvements were not observed for the older children. The improvements in the younger children may be explained by a higher disease burden, more parental oversight, and increased use of the at-home gamified spirometer. Overall, the participants demonstrated an increase in lung function. Further studies are needed to confirm these findings and assess the long-term benefits.

## Data Availability

Qualified researchers may request access to patient level data and related study documents including the study protocol and the statistical analysis plan. Requests will be assessed for scientific merit, product approval status, and conflicts of interest. If the request is approved, patient level data will be de-identified and study documents will be redacted to protect the privacy of trial participants and to protect commercially confidential information. Please email USMedInfo@tevapharm.com to make your request.
