# Supplementary material for: Use of a digital rescue inhaler and at-home spirometer among inner-city children with asthma: a real-world experience
Source: Front Allergy. 2025 Sep 22;6:1641312. doi: 10.3389/falgy.2025.1641312 (PMC12497696; doi:10.3389/falgy.2025.1641312)
Supplement: Supplementary file 1 [file Table1.docx]

| **Supplementary Table 1: Digihaler Rescue Inhaler Use** | | | | | | |
| --- | --- | --- | --- | --- | --- | --- |
| **Participant** | **Total fair/good inhalations** | **Total study days** | **Inhalations per week** | **Rescue free days** | **Estimated non-Digihaler rescue use** | **Reason for non-Digihaler rescue use** |
| 1 | 41 | 139 | 2.1 | 81% | 0 |  |
| 2 | 4 | 113 | 0.2 | 96% | 0 |  |
| 3 | 17 | 120 | 1.0 | 96% | **50%** | Preferred HFA |
| 4 | 127 | 126 | 7.1 | 29% | 0 |  |
| 5 | 12 | 134 | 0.6 | 97% | **80%** | Did not take Digihaler |
| 6 | 38 | 127 | 2.1 | 57% | 0 |  |
| 7 | 287 | 78 | 25.8 | 6% | 0 |  |
| 8 | 335 | 98 | 23.9 | 2% | 0 |  |
| 9 | 1 | 106 | 0.1 | 99% | **20%** | Preferred nebulizer |
| 10 | 5 | 122 | 0.3 | 98% | 0 |  |
| 11 | 18 | 119 | 1.1 | 92% | 0 |  |
| 12 | 1 | * | * |  |  |  |
| 13 | 353 | 85 | 29.1 | 54% | 0 |  |
| 14 | 41 | 148 | 1.9 | 72% | 0 |  |
| 15 | * | 88 | * |  |  |  |
| 16 | * | 88 | * |  |  |  |
| 17 | 7 | 75 | 0.7 | 96% | 0 |  |
| 18 | 96 | 113 | 5.9 | 82% | **26%** | Given in clinic or ER |
| 19 | 201 | 91 | 15.5 | 54% | 0 |  |
| 20 | 146 | 92 | 11.1 | 26% | 0 |  |
| 21 | 4 | 98 | 0.3 | 98% | 0 |  |
| **Mean (SD)** | **91 (119)** | **108 (21)** | **7.1 (9.8)** | **69% (33%)** | **8% (20%)** |  |
| * = No record or no post-visit for Digihaler inhalations | | | | | | |

| **Supplementary Table 2 – Proper step identification for rescue inhaler technique by survey**  **[Data as n (%) unless otherwise specified]** | | |
| --- | --- | --- |
|  | **Baseline** | **Post** |
| **Nebulizer use (n)** | **10** | **6** |
| **HFA Inhaler use** |  |  |
| **Spacer (n)** | **19** | **15** |
| Yes**^†^** | 10 (53%) | 4 (27%) |
| No | 9 (47%) | 11 (73%) |
| **Breaths/puff (n)** | **19** | **15** |
| Take a breath after each puff**^†^** | 18 (95%) | 14 (93%) |
| Take a breath after all puffs | 1 (5%) | 1 (7%) |
| **Breathing speed (n)** | **9** | **11** |
| One fast short breath in | 0 (0%) | 0 (0%) |
| Slow long breath**^†^** | 9 (100%) | 11 (100%) |
| **Breathing technique (n)** | **10** | **4** |
| Slow long breath in and hold 10 seconds**^†^** | 8 (80%) | 4 (100%) |
| Wait until I take 6-10 regular breaths**^†^** | 1 (10%) | 0 (0%) |
| Wait until I take 1-5 regular breaths | 1 (10%) | 0 (0%) |
| **Overall correct steps identified** | **76%** | **73%** |
| **Powder Inhaler use** |  |  |
| **Set-up (n)** | **2** | **9** |
| Hold inhaler upright the whole time**^†^** | 1 (50%) | 5 (56%) |
| Open cap down until click for each breath**^†^** | 2 (100%) | 6 (67%) |
| Breathe all air out**^†^** | 0 (0%) | 5 (56%) |
| Close lips tightly around device for each breath**^†^** | 2 (100%) | 5 (56%) |
| Make sure not to block vent with lips/fingers**^†^** | 1 (100%) | 5 (56%) |
| **Breathing technique (n)** | **2** | **9** |
| Breathe in quickly/deeply | 1 (50%) | 0 (0%) |
| Breathe in quickly/deeply and hold 10 seconds**^†^** | 1 (50%) | 3 (33%) |
| Breathe in long and slow | 0 (0%) | 2 (22%) |
| Breathe in long and slow and hold 10 seconds | 0 (0%) | 4 (44%) |
| **Overall correct steps identified** | **64%** | **54%** |
| **Pre-treatment use (n)** | **12** | **12** |
| 1-10 minutes before exercise | 6 (50%) | 6 (50%) |
| **10-30 minutes before exercise^†^** | **6 (50%)** | **5 (42%)** |
| 30-60 minutes before exercise | 0 (0%) | 1 (8%) |
| >60 minutes before exercise | 0 (0%) | 0 (0%) |
| Only after starting exercise | 0 (0%) | 0 (0%) |
| † = denotes correct method | | |

| **Supplementary Table 3: Aluna at-home Gamified Spirometer Use** | | | | |
| --- | --- | --- | --- | --- |
| **Participant** | **Total Spirometry uses** | **Total days Spirometer was used** | **Total study days** | **Average Spirometer use per study day** |
| 1 | 2 | 2 | 139 | 0.01 |
| 2 | 11 | 4 | 113 | 0.10 |
| 3 | 28 | 25 | 120 | 0.23 |
| 4 | 6 | 3 | 126 | 0.05 |
| 5 | 0 | 0 | 134 | 0.00 |
| 6 | 6 | 3 | 127 | 0.05 |
| 7 | 1 | 1 | 78 | 0.01 |
| 8 | 124 | 36 | 98 | 1.27 |
| 9 | 0 | 0 | 106 | 0.00 |
| 10 | 84 | 70 | 122 | 0.69 |
| 11 | 106 | 84 | 119 | 0.89 |
| 12 | 10 | 4 | * |  |
| 13 | 4 | 4 | 85 | 0.05 |
| 14 | 192 | 113 | 148 | 1.30 |
| 15 | 7 | 7 | 88 | 0.08 |
| 16 | 10 | 9 | 88 | 0.11 |
| 17 | 126 | 48 | 75 | 1.68 |
| 18 | 17 | 4 | 113 | 0.15 |
| 19 | 25 | 22 | 91 | 0.27 |
| 20 | 44 | 39 | 92 | 0.48 |
| 21 | 13 | 2 | 98 | 0.13 |
| **Mean (SD)** | **39 (54)** | **23 (32)** | **108 (21)** | **0.38 (0.51)** |
| * = No post-visit, not included in mean calculation | | | | |

| **Supplementary Table 4 – Asthma management and reported risk** | | | | | | | | | | |  |  |
| --- | --- | --- | --- | --- | --- | --- | --- | --- | --- | --- | --- | --- |
| **Initial Visit** |  |  |  |  | **Post Visit** |  |  |  | **Overall Controller Increase** | **Overall Controller Decrease** |  | |
| **Participant** | **Treatment** | **Controller** | **Dose** | **Adherence** | **Treatment** | **Controller** | **Dose** | **Adherence** |  |  | **Missed School (days)** | **ER & Urgent Care (visits)** |
| 1 | None |  |  |  | Controller | Wixela 100/50 | 1 inhalation twice daily | Almost Never | ● |  | 0 | 0 |
| 2 | Rescue only |  |  |  | Rescue only |  |  |  |  |  | 0 | 0 |
| 3 | Controller | Inhub 250/50 | 2 puffs once daily | Always | Rescue only |  |  |  |  | ● | 1 | 0 |
| 4 | Rescue only |  |  |  | None |  |  |  |  | ● | 1 | 0 |
| 5 | Controller | Airduo 232/14 | 1 inhalation twice daily | Always | Rescue only |  |  |  |  | ● | 0 | 0 |
| 6 | Controller | Qvar 80 | 2 inhalations twice daily | Always | Rescue only |  |  |  |  | ● | 0 | 2 |
| 7 | Controller | Wixela (250/50) | 2 inhalations twice daily | Almost always | Controller | Wixela 250/50 | 2 inhalations twice daily | Almost always |  | ● | 0 | 0 |
|  | Montelukast |  |  | Almost always |  |  |  |  |  |  | 0 | 0 |
| 8 | Controller | Wixela 250/50 | 1 inhalation once daily | Almost always | Controller | Wixela 250/50 | 1 inhalation once daily | Almost always |  |  | 2 | 2 |
| 9 | Rescue only |  |  |  | Rescue only |  |  |  |  |  | 0 | 0 |
| 10 | Controller | Qvar 40 | 2 inhalations twice daily | Almost always | Controller | Qvar 40 | 2 inhalations twice daily | Almost always |  |  | 0 | 0 |
| 11 | Controller | Qvar40 | 1 inhalation once daily | Always | Rescue only |  |  |  |  | ● | 12 | 0 |
| 12 | Rescue only |  |  |  | *No Post Visit* |  |  |  |  |  | 0 | 0 |
| 13 | Controller 1 | Advair 230/21 | 2 puffs twice daily | Almost always | Controller 1 | Advair 230/21 | 2 puffs twice daily | Almost always | ● |  | 2 | 2 |
|  | Controller 2 | Spiriva Respimat 1.25 | 2 inhalations once daily | Almost always | Controller 2 | Spiriva Respimat 1.25 | 2 inhalations once daily | Almost always |  |  | 0 | 0 |
|  |  |  |  |  | Biologic | Dupilumab | 600mg loading (12 days prior) |  |  |  | 0 | 0 |
| 14 | Controller | Symbicort 160/4.5 | 2 puffs twice daily | Almost always | Controller | Symbicort 160/4.5 | 2 puffs twice daily | Most days |  | ● | 0 | 2 |
|  | Montelukast |  |  | Always | Montelukast |  |  | Most days |  |  | 0 | 0 |
| 15 | None |  |  |  | None |  |  |  |  |  | 2 | 0 |
| 16 | Rescue only |  |  |  | None |  |  |  |  | ● | 0 | 2 |
| 17 | Controller | Symbicort 160/4.5 | 2 puffs twice daily | Almost always | Controller | Breo 200/25 | 1 inhalation once daily | Always | ● |  | 0 | 0 |
|  | Montelukast |  |  | Almost always | Montelukast |  |  | Almost always |  |  | 0 | 0 |
| 18 | Controller | Advair 250/50 | 1 inhalation once daily | Some days | Controller | Advair 250/50 | 1 inhalation once daily | Some days |  |  | 0 | 0 |
| 19 | Controller | Qvar 80 | 2 puffs once daily | Almost always | Controller | Qvar 80 | 2 puffs once daily | Almost always |  |  | 1 | 0 |
| 20 | Controller | Flovent 44 | 2 puffs twice daily | Almost always | Rescue only |  |  |  |  | ● | 1 | 0 |
| 21 | Controller | Qvar 40 | 2 inhalations once daily | Almost never | Controller | Symbicort 80/4.5 | 2 puffs once daily | Almost never | ● |  | 0 | 0 |
|  | | | | | | | | | | | | |
|  | | | | | | | | | | |  |  |
